# COVID‐19 and cardiac surgery: A perspective from United Kingdom

**DOI:** 10.1111/jocs.15039

**Published:** 2020-09-27

**Authors:** Amer Harky, Deborah Harrington, Omar Nawaytou, Ahmed Othman, Catherine Fowler, Gareth Owens, Francesco Torella, Manoj Kuduvalli, Mark Field

**Affiliations:** ^1^ Department of Cardiac Surgery Liverpool Heart and Chest Hospital Liverpool UK; ^2^ School of Medicine, Faculty of Health and Life Sciences University of Liverpool Liverpool UK; ^3^ Liverpool Centre for Cardiovascular Science University of Liverpool and Liverpool Heart and Chest Hospital Liverpool UK; ^4^ Aortic Dissection Awareness UK & Ireland; ^5^ Liverpool Vascular and Endovascular Service Royal Liverpool University Hospital Prescot Street Liverpool UK; ^6^ School of Physical Sciences University of Liverpool Liverpool UK

**Keywords:** cardiac surgery, COVID‐19, NHS, service, United Kingdom

## Abstract

The emergence of severe acute respiratory syndrome coronavirus 2 in December 2019, presumed from the city of Wuhan, Hubei province in China, and the subsequent declaration of the disease as a pandemic by the World Health Organization as coronavirus disease 2019 (COVID‐19) in March 2020, had a significant impact on health care systems globally. Each country responded to this disease in different ways, however this was done broadly by fortifying and prioritizing health care provision as well as introducing social lockdown aiming to contain the infection and minimizing the risk of transmission. In the United Kingdom, a lockdown was introduced by the government on March 23, 2020 and all health care services were focussed to challenge the impact of COVID‐19. To do so, the United Kingdom National Health Service had to undergo widespread service reconfigurations and the so‐called “Nightingale Hospitals” were created de novo to bolster bed provision, and industries were asked to direct efforts to the production of ventilators. A government‐led public health campaign was publicized under the slogan of: “Stay home, Protect the NHS (National Health Service), Save lives.” The approach had a significant impact on the delivery of all surgical services but particularly cardiac surgery with its inherent critical care bed capacity. This paper describes the impact on provision for elective and emergency cardiac surgery in the United Kingdom, with a focus on aortovascular disease. We describe our aortovascular activity and outcomes during the period of UK lockdown and present a patient survey of attitudes to aortic surgery during COVID‐19 pandemic.

## 1 BACKGROUND TO COVID‐19 AND EFFECTS ON SERVICES FOR CARDIAC SURGERY IN THE UNITED KINGDOM

**Figure 1:**
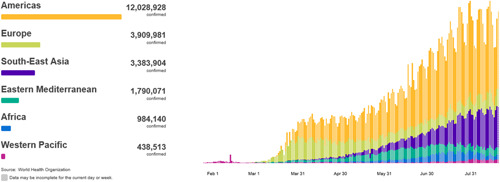
World Health Organization statistics of coronavirus disease 2019 globally. *Source*: www.WHO.int (Accessed August 22^nd^, 2020)

**Figure 2:**
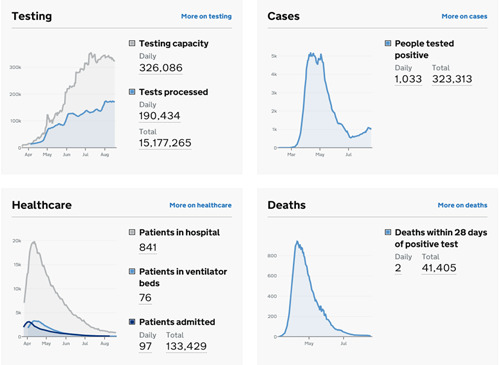
United Kingdom coronavirus disease 2019 status of confirmed cases and deaths. *Source*: www.gov.uk (Accessed August 22^nd^, 2020)

The severe acute respiratory syndrome coronavirus 2 first emerged in the city of Wuhan, China, in December 2019 and it has since spread rapidly across the globe, causing a disease named as coronavirus disease 2019 (COVID‐19) in February 2020 and with the World Health Organization declaring a pandemic in March 2020.^1^ As of August 20^th^, 2020, there were more than 22.2 million confirmed cases of COVID‐19 and over 795,000 deaths reported globally (Figure 1) with the United Kingdom being 12th among countries in term of confirmed cases (more than 326,000) and 5th in COVID‐19‐related deaths (more than 41,000; Figure 2).^2^

**Figure 3:**
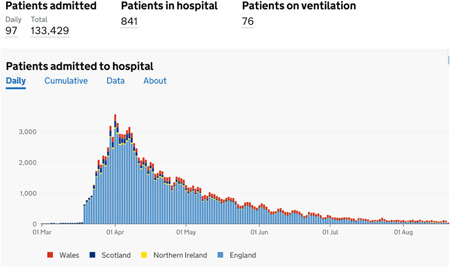
Status of patient admission to hospital and requirement of mechanical ventilation in coronavirus disease 2019 patients in the United Kingdom. *Source*: www.gov.uk (Accessed August 22, 2020)

Public Health England (PHE) published the very first report on COVID‐19 on January 22, 2020. Just 1 day later, the Emergency Department at Royal London Hospital swabbed its first potential COVID‐19 patient.^3^ The declaration of this disease as a pandemic put health care systems in the United Kingdom on alert and the government introduced a national lockdown on March 23, 2020 in an attempt to contain the disease and minimize the transmission risk to others. A campaign was launched under the slogan of “Stay home, Protect the NHS (National Health Service) and Save lives.” Although a critical step to combat this highly contagious disease, it created a significant burden on an otherwise freely accessible health care system, the NHS. The NHS had to undergo a significant transformation diverting resources to frontline health care services including ambulance services, emergency departments, and allocation of intensive care beds in preparation for the potential influx of COVID‐19 patients and the requirement for ventilatory support (Figure 3). De novo facilities, the Nightingale Hospitals, were created throughout the nation to increase capacity; private hospital capacity was purchased, industries were tasked with producing ventilators and academia with producing treatments and vaccines. Effectively, all elective care was stopped with services only maintained for emergencies.

Amongst the many specialties affected by the NHS service reconfiguration was cardiac surgery, given its ownership of a large resource of ventilated beds normally required in elective practice. Attempts were made in a number of regions to create centralized cardiac surgical services to continue the provision of care to this high‐risk cohort and avoid secondary deaths due to untreated cardiovascular diseases.^4^ The Royal College of Surgeons (RCS), Society of Cardiothoracic Surgery in UK and Ireland (SCTS), and the NHS issued guidelines and regular updates on the practice of cardiac surgery during this pandemic, introducing protocols and pathways to minimize the risk of COVID‐19 to patients and staff without affecting the quality of service and care to those needing cardiac surgery.^5^, ^6^, ^7^, ^8^, ^9^, ^10^, ^11^

The network of centers that perform cardiac surgery in England generally responded to the crisis according to government guidance by reducing or, more frequently, halting elective operating, but with a degree of independence. The exact timeline during which each center wound down elective and urgent services varied according to local circumstances and pressures. The processes by which each center managed patient pathways were dependent on local arrangements. In addition, England, Wales, Scotland, and Northern Ireland, each with its own devolved government, responded differently. This paper focusses on the experience of Liverpool Heart and Chest Hospital (LHCH) with changes to service provision for cardiac surgery, focussing on aortovascular patients.

## 2 RECOMMENDATIONS AND GUIDELINES DURING COVID‐19 IN BRITAIN

On March 20, 2020, the RCS published its initial, brief guidance for surgeons who were working during the COVID‐19 pandemic, emphasizing the safety of the working force as well as the maintenance of emergency surgical workforce and capabilities.^5^ The detailed guidance came into force on March 26^th^, 2020 outlining the scope of patient selection and flow of surgical practice across the United Kingdom. Since then, the guidelines have been updated four times, lastly on June 5^th^, 2020.

The initial guidance involved the cancellation of all elective operating cases, with a focus on operating on urgent/emergency and otherwise life‐saving procedures.^6^ Patients were categorized into four levels according to their need for surgery:

- Priority level 1a Emergency—operation needed within 24 h.
- Priority level 1b Urgent—operation needed with 72 h.
- Priority level 2 Surgery that can be deferred for up to 4 weeks.
- Priority level 3 Surgery that can be delayed for up to 3 months.
- Priority level 4 Surgery that can be delayed for more than 3 months.

With the gradual decline in the cases of COVID‐19, the service gradually resumed its activities, slowly reintroducing elective surgery on a phased basis. Elective cases were prioritized as Red, Amber, Green (RAG rating) with red been classified as “urgent elective.”

With a similar approach but at a more specialized level, the SCTS introduced national guidelines on the performance of cardiac surgery. As its initial response, the society introduced a clear cardiothoracic surgery escalation framework on March 16^th^ and 18^th^, 2020; which outlined the routine practice of operating theaters, clinics, and the running of a multidisciplinary team (MDT) meetings.^7^ It classified cardiothoracic patients in four areas, the obligatory in‐patients, which required surgical intervention, the alternative (nonsurgical) pathways including inpatients and those to be managed by ambulatory base services, the day‐cases, and finally, the outpatients, whose hospital visits were to be kept at the minimum safe level. The society also developed a clear pathway for patient selection during the initial lockdown and to smooth the gradual resumption of elective activity. The guidelines not only included patient selection but also focused on triage methods of such cohort, COVID‐19 screening methods and tests, the use of personal protection equipment, and the management of operating theaters. These guidelines were implemented nationwide and helped in containing the spread of COVID‐19 in cardiac surgery patients.^8^ The society's latest guideline on resumption of elective activity eliminates the requirement for preoperative radiological screening if they have been self‐isolating for 14 days before surgery, provided that they have no COVID‐19‐related symptoms and have negative COVID‐19 nasopharyngeal swab within 72 h of surgery date.^9^

The NHS also issued several, nationwide guidelines to provide insights on speciality practice during the COVID‐19 pandemic. Most of the clinical guidelines and recommendations were interlinked with the work of the RCS and SCTS. The NHS and PHE recognized that cardiothoracic surgery, like any other speciality, needed service modification which depended on the unit and the region of service, considering that some cardiothoracic units are incorporated as part of large trauma centers while others are tertiary units without emergency department service.^10^ The NHS categorized the patients into six major groups:

1. Obligatory in‐patients: Those patients who need immediate admission and surgical intervention.
2. Alternative pathways: This is categorized into two subgroups:
  a. *In‐patient*: The condition can reasonably be managed on an ambulatory basis after a more limited in‐patient stay than normal; for example, ambulatory chest drain management.
  b. *Ambulatory*: The condition can reasonably be managed on an ambulatory basis.
3. Day‐cases: Surgery can be safely undertaken for a large number of conditions.
4. Surgery and interventional care that can be postponed.
5. Trauma surgery.
6. First contact and clinics.

In addition to the above, the work of the cardiothoracic team was expanded to have a consultant‐led service, including patient assessment, daily reviews, and decision‐making processes. The NHS also advised to restructure training and education needs during this time period to give priority to COVID‐19 patient care provision.^12^, ^13^ In its latest guide, the NHS advised to utilize a remote consultation, where appropriate. However, when face‐to‐face consultations were needed, patients were brought in for further assessment in a controlled and organized manner.^11^

PHE, NHS, SCTS, and RCS eventually merged their statements to restructure the daily practice of cardiac surgery including modification of hospital setups, patient selection, and screening process as well as standards for intubation, operating, and provision of perioperative care for such patients. The joint statements were released in accordance with the severity of the COVID‐19 pandemic within the UK general population and the phase of the disease.

## 3 SERVICE TRANSFORMATION

The NHS has been stretched to provide care for the already aging population alongside the new cases of infected COVID‐19. As such and due to limited capacity, there have been some attempts at the reconfiguration of services, in some regions, by creating centralized units to provide care for subspecialities that are not in direct response to COVID‐19. This service modification entailed the creation of detailed and tailor‐made protocols for planning cardiac surgery whilst optimizing the use of intensive care and ward beds for the treatment of COVID‐19 cases. Such a process required a nationwide assessment of capacity and capabilities to accommodate such changes. In the North‐West of England, which serves a population of 7.3 million, cardiac care was channeled through four major cardiothoracic units: Blackpool, Manchester Royal, Manchester Wythenshawe, and LHCH; LHCH was chosen to be the central unit for cardiac and aortic surgery and led the development of the North‐West Urgent Cardiothoracic Service (NUCS) Protocol to guide patient treatment pathways (Appendix 1). As NUCS was set up, government measures took effect, reducing admissions; in reality, few patients were channeled into Liverpool from other cardiac units, but some throughput continued from our usual catchment area. North‐West regional pathways still exist in preparation for a potential second spike. Similarly, in London the service was reconfigured to operate in only two units among the combined seven NHS centers serving the population of 8.5 million people, forming the Pan London Emergency Cardiac Surgery (PLECS) service.^14^ It is important to emphasize that the base of developing such centralized services and the detailed protocol was to provide a COVID‐19 free environment for patients undergoing cardiac surgeries. This is a very critical point as COVID‐19 seems to have a significant correlation with cardiovascular diseases and outcomes.^15^, ^16^, ^17^

**Figure 4:**
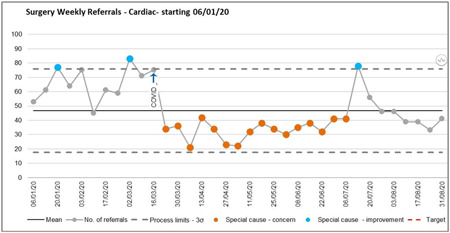
Average weekly cardiac surgery activities at Liverpool Heart and Chest Hospital

Maintenance of a COVID‐19 free environment with clean patient pathways was key to maintaining a limited but safe service. There was a significant reduction in the operational activities, as high as 83% in some cardiac surgical units.^4^ Our center observed similar reductions (Figure 4). Eventually, the establishment of standardized patient pathways (Appendix 2) for perioperative care and management in the theater (Appendix 3) aided in a gradual increase in surgical activities. According to regional pathways (NUCS and PLECS), patients were classified into four major categories:

- Level 1: Elective patients who have indications for routine cardiac surgery and would normally be added to an elective waiting list.
- Level 2: Urgent patients at home who are on the existing waiting lists or in the process of referral but have critical/life‐threatening anatomy with worsening symptoms or the need for urgent prognostic intervention.
- Level 3: Urgent patient undergoing interhospital transfers who by definition are in hospital with prognostic/critical anatomy or physiology or with unstable symptoms. They require cardiac surgery within this hospital admission (but not on the same day), and no other options for treatment are possible such as medical or percutaneous intervention.
- Level 4: Emergency cases which are most commonly acute aortic dissections, such patients have life‐threatening emergency aortic and cardiac conditions and require surgery within hours.

For NUCS the decision‐making process started with the receipt of an urgent inpatient referral, after triage at the referring regional cardiac hospital (Blackpool and Manchester). These were directed to our local COVID‐19 daily MDT along with our local urgent referrals. All our 10 weekly MDTs were amalgamated into a single and virtual COVID‐19 MDT with widespread attendance. After review of the available information, an outcome was communicated to the referring clinical team and the patient. If the intervention was deemed necessary, then procedural planning took place and the case was allocated to a consultant and the date for surgery identified. Emergency referrals were processed in the usual way by on‐call staff. A number of patients requiring emergency care were referred to Liverpool on the basis of the NUCS arrangement.

## 4 AORTOVASCULAR DISEASE AND COVID‐19 AT LHCH

LHCH is one of the very few centers in the United Kingdom offering medical and surgical services for patients with complex aortovascular diseases. The hospital is the only stand‐alone Trust in the United Kingdom offering only cardiovascular and thoracic services and as such has no Emergency Department or Acute Medical Admissions Facility. Four of fifteen cardiac surgeons specialize in aortovascular surgery with a separate emergency on‐call rota. The team also works with local vascular surgeons under the banner of Liverpool Cardiovascular Surgery, with regular joint operating, commonly on hybrid cases. From March 23, the independent elective listing of patients for surgery by consultants was abandoned. General cardiac activity was wound down, under the direction of the central government, to free up critical care capacity for the potential transfer of COVID‐19 patients from acute hospitals in the region. The activity was reduced from five cardiac theaters and 10 cases per day to four theaters and four cases per day, with only urgent patients allocated from a common pool. Aortovascular patients, urgent and emergency, had to compete with cardiac surgical patients for theater space. All patients were discussed at the daily virtual “COVID‐19 MDT” where an emphasis was placed on directing patients towards medical or minimally invasive therapy (endovascular) whenever possible. With time, a number of high‐risk elective patients were operated.

### 4.1 Risk assessment of elective aortovascular patients

A major issue in this period was the quantification of postoperative COVID‐19 infection in “clean” patients, thus balancing the additional risks of death from viral infection versus the risk of a putative delay in surgery—a delay of at least 3 months was presumed. For aortovascular disease, the Vascular Society of Great Britain & Ireland (UK) offered guidance by increasing the size threshold for elective intervention for the abdominal aortic aneurysm to more than 7 cm^18^ as did the Society for Vascular Surgery in the United States, recommending intervention only on the symptomatic thoracoabdominal disease.^19^ The evidence base underlying this advice was opaque at best. We “RAG rated” (Red, Amber, Green) and chose to operate on the so‐called “Red urgent elective” patients with COVID screening and “clean hospital pathways.” The definition of red was a symptomatic severe disease. During this period, we made no adjustments to size‐based guidelines.

### 4.2 Emergency aortovascular patients

There were unanimous recommendations from all advisory groups to treat emergency life‐threatening disease as normal while adopting appropriate safeguarding procedures for staff and other patients within the hospital.

### 4.3 Referral activity

A commonly observed phenomenon during this period was a dramatic reduction in both elective and urgent/emergency referrals thought to be due to very few patients presenting to the hospital due to a fear of COVID‐19 and local triage by referring doctors.

### 4.4 Outcomes of operated aortovascular patients

We examined our outcomes between the dates of March 1, 2020 and July 4, 2020. A total of 59 patients were operated (Table 1) during this period. In normal times we would expect the four aortovascular surgeons to perform roughly one elective/urgent case each per week over 42 weeks/year (i.e., total 56 cases) plus emergencies, suggesting our aortovascular activity was largely maintained during this 14‐week period.

(i) *Elective (Red on RAG rated)*: During this period, we performed operations on elective patients including root, arch, descending thoracic aorta, and thoracoabdominal aortic aneurysm surgery including thoracic endovascular aortic repair. One of these elective patients turned COVID‐19 positive in the postoperative period but did not develop COVID‐19 pneumonia; the COVID‐19‐related mortality was zero.
(ii) *Urgent*: Urgent patients were those referred in from other hospitals and in‐house patients requiring surgery during the same admission. Patients were screened for COVID‐19 at referring hospitals and underwent computed tomography (CT) screening and repeat COVID‐19 swabs, lactic dehydrogenase assay, and lymphocyte measurements on transfer. We operated on 21 such patients. None developed COVID‐19 but there were three deaths.
(iii) *Emergency*: Emergency patients came into our unit from referring hospitals and were taken to the theater immediately with COVID‐19 status unknown. We operated on nine such patients, two of whom developed COVID‐19 in the postoperative course but not COVID‐19 pneumonia. There was one non‐COVID‐19‐related death.
(iv) *Medically managed patients*: We managed 15 aortovascular patients without surgery either because it was not indicated or because patients were unfit for the necessary surgical procedure. Eight were type A dissections (moribund, 3; major stroke, 1; subacute, 1; or patient too frail/comorbid; 3). Five patients had surgically relevant thoracoabdominal aortic dissection or aneurysm but were too frail/comorbid; one was an uncomplicated acute type B (COVID‐19 positive). One patient had a root abscess that was COVID‐positive and died while awaiting a negative swab before transfer.

**Table 1:** Perioperative characteristics of patients that underwent aortovascular intervention at Liverpool Heart and Chest Hospital between March 1, 2020 and July 3, 2020

Variable	Total (*n* = 59)	Elective (*n* = 29)	Urgent (*n* = 21)	Emergency (*n* = 9)
Preoperative				
Mean age (*SD*)	61.3 ± 14	65.0 ± 13.9	59.4 ± 13.9	53.4 ± 11.5
Male (%)	39 (66)	15 (52)	18 (86)	6 (67)
HTN (%)	36 (61)	20 (69)	11 (52)	5 (56)
Diabetes mellitus (%)	4 (7)	3 (10)	1 (5)	0 (0)
COPD (%)	7 (12)	4 (14)	3 (14)	0 (0)
Creatinine (*SD*)	81 ± 24	73 ± 21	82 ± 22	101 ± 37
PVD (%)	2 (3)	1 (3)	1 (5)	0 (0)
NYHA class III–IV (%)	30 (51)	11 (38)	15 (71)	4 (44)
Previous cardiac surgery (%)	7 (12)	3 (10)	4 (19)	0 (0)
Previous aortic surgery (%)	13 (22)	6 (20)	6 (29)	1 (11)
Previous endovascular intervention (%)	1 (2)	1 (3)	0 (0)	0 (0)
BAV (%)	10 (17)	3 (10)	5 (24)	2 (22)
Marfan (%)	5 (9)	3 (10)	2 (10)	0 (0)
COVID‐19 status				
Preoperative				
Negative (%)	44 (75)	20 (69)	18 (86)	6 (67)
Positive (%)	0 (0)	0 (0)	0 (0)	0 (0)
Unknown (%)	15 (25)	9 (31)	3 (14)	3 (33)
LDH (*SD*)	201 ± 73	181 ± 41	229 ± 12	na
Lymphocyte count (*SD*)	1.22 ± 0.51	1.21 ± 0.38	1.29 ± 0.61	1.31 ± 0.50
CT thorax (%)	36 (66)	19 (66)	16 (76)	2 (20)
Postoperative				
Negative (%)	56 (95)	28 (97)	21 (100)	7 (78)
Positive (%)	3 (5)	1 (3)	0 (0)	2 (22)
COVID pneumonia (%)	0 (0)	0 (0)	0 (0)	0 (0)
COVID‐related death (%)	0 (0)	0 (0)	0 (0)	0 (0)
Non‐COVID‐related death (%)	4 (6)	0 (0)	3 (14)	1 (11)
Pathology				
Aneurysm (%)	39 (66)	25 (86)	13 (62)	1 (11)
Aortic dissection (%)	12 (21)	4 (14)	2 (10)	6 (67)
IMH (%)	2 (3)	0 (0)	0 (0)	2 (22)
Others (%)	6 (10)	0 (0)	6 (28)	0 (0)
Operative				
Isolated root (%)	19 (31)	6 (20)	7 (33)	6 (67)
Hemi arch (%)	11 (18)	3 (10)	3 (14)	5 (56)
Total arch (%)	4 (6)	2 (7)	0 (0)	2 (22)
FET (%)	4 (6)	2 (7)	0 (0)	2 (22)
DTA (%)	10 (16)	7 (24)	3 (14)	0 (0)
TAAA (%)	10 (16)	6 (20)	4 (19)	0 (0)
TEVAR (%)	5 (8)	3 (14)	1 (5)	1 (10)
Postoperative				
Mechanical ventilation time (h,*SD*)	27 ± 33	25 ± 33	23 ± 30	44 ± 33
Length of ICU stay (h, *SD*)	126 ± 125	113 ± 132	111 ± 100	155 ± 125
Tracheostomy (%)	3 (5)	1 (3)	1 (5)	1 (10)
Reoperation for bleeding (%)	8 (13)	5 (17)	2 (10)	1 (10)
Mesenteric ischemia (%)	1 (2)	0 (0)	1 (5)	0 (0)
Stroke (%)	6 (10)	3 (10)	2 (10)	1 (10)
Paraplegia (%)	2 (3)	1 (3)	1 (5)	0 (0)
Renal replacement therapy (%)	5 (8)	3 (10)	2 (10)	0 (0)
Acute MI (%)	0 (0)	0 (0)	0 (0)	0 (0)
30‐Day mortality postopen repair (%)	2 (3)	0 (0)	2 (10)	0 (0)
30‐Day mortality post‐TEVAR (%)	2 (3)	0 (0)	1 (5)	1 (10)

Abbreviations: BAV, bicuspid aortic valve; COPD, chronic obstructive pulmonary disease; COVID‐19, coronavirus disease 2019; CT, computed tomography; DTA, descending thoracic aorta; FET, frozen elephant trunk; HTN, hypertension; ICU, intensive care unit; IMH, intramural hematoma; LDH, lactate dehydrogenase; MI, myocardial infarction; NYHA, New‐York Heart Association; PVD, peripheral vascular disease; TAAA, thoracoabdominal aortic aneurysm; TEVAR, thoracic endovascular aortic repair.

No patient in this cohort died of postoperative COVID‐19 pneumonia. It should be noted that our critical care area is divided into four distinct rooms, an arrangement that facilitated the isolation of COVID‐19‐positive patients. During this period, we regularly admitted ventilated patients from neighboring acute hospitals with community‐acquired COVID‐19. In summary, we attempted to maintain our aortovascular patients COVID‐19‐free via a combination of preoperative screening, strict theater procedures, and separate pathways the “clean” and the COVID‐19 cohort (Appendces 1–3).

It should be noted that our preoperative screening protocols changed as evidence presented itself. At the start of the lockdown period, we performed routine CT scanning and bronchoalveolar lavage (BAL) in theater or when a patient returned to intensive treatment unit. During late June 2020, we eventually abandoned CT scanning and a plain chest radiograph was used instead to identify individuals with early or suspected COVID pneumonia. In addition, it became clear the BAL was highly sensitive in the detection of viral RNA, but it was unclear whether this was simply dead virus indicating previous exposure or rather an active infection. Our experience showed that a positive BAL was of no consequence for the clinical course of the patient but created major issues for bed capacity with a need for isolation. For this reason and during late July, BAL was stopped in elective patients with a preoperatively negative COVID‐19 swab, normal chest X‐ray, and blood tests who had been isolating for 2 weeks.

We are thus only aware of one patient who should have undergone urgent surgery for a root abscess but died following delays while awaiting his status to change from COVID‐19 positive to negative. To our knowledge, no patients came to harm while on our waiting lists for delayed elective surgery. We see this as validation of the systems we developed to balance the need to make our critical care beds available for the national COVID‐19 pandemic and the needs of our patients with life‐threatening cardiovascular disease.

After this period, we gradually returned to normal work patterns, with surgeons planning their operating lists independently, progressively increasing elective activity as hospital pathways allowed. We still use a RAG rating system at present.

## 5 PATIENT PERSPECTIVE

To better understand the beliefs and attitudes of patients with aortic pathology during COVID‐19, we conducted a survey through Aortic Dissection Awareness UK and Ireland (ADA UKI).

A structured questionnaire was developed and preoperative patients who are members of ADA UKI were invited to complete it from August 17 and 25, 2020. A total of 29 responses were received and the results are presented in Table 2.

**Table 2:** Results of survey questions among patients with aortic pathologies

Question	Yes	No
Which of the following best describes you		
*I am currently being considered for or awaiting aortic surgery*	1/29 (3%)	
*I am under regular surveillance for aortic disease and may need surgery in the future*	28/29 (97%)	
Are you concerned about attending the hospital for aortic surgery during the COVID‐19 pandemic?	8/29 (28%)	21/29 (72%)
Are you confident that your hospital will be able to greatly reduces the risk of you catching COVID‐19 after your operation?	23/29 (79%)	6/29 (21%)
Which of the following are you most concerned about?		
*The risk to your life from your aortic disease*	24/29 (83%)	
*The risk of catching COVID‐19 in hospital*	5/29 (17%)	
Taking into account the risk to your life from your aortic disease and the risk of catching COVID‐19, would you prefer to delay your surgery until there is a vaccine for COVID‐19?	8/29 (28%)	21/29 (72%)
Are you concerned about attending the hospital for a follow‐up scan for your aortic disease during the COVID‐19 pandemic?	7/29 (24%)	22/29 (76%)
Which of the following would you prefer?		
*My regular follow‐up scans to be delayed until there is a COVID‐19 vaccine*	3/29 (10%)	
*My regular follow‐up scans to begin as soon as possible despite COVID‐19*	26/29 (90%)	
How would you prefer to discuss your follow‐up scans?		
*Face‐to‐face, with a doctor in the clinic at the hospital*	17/29 (59%)	
*Virtually, with a doctor over the telephone*	12/29 (41%)	
Do you think that COVID‐19 poses additional risk to you because you have Aortic disease?	23/29 (79%)	6/29 (21%)
If you developed chest pain, would you be willing to go to the hospital immediately?	26/29 (90%)	3/29 (10%)

Abbreviation: COVID‐19, coronavirus disease 2019.

Among the 29 patients, only one of them considered himself as “awaiting surgery” while the rest 28 patients considered themselves as “under surveillance” although they have been offered the option of surgical intervention at the time of first assessment and rather awaiting a confirmed date for surgery which has been significantly affected by COVID‐19 pandemic.

More than 80% of them were more concerned about the delayed aortic surgery than the possibility of contracting COVID‐19 in‐hospital; over 70% of them had no concerns in attending hospital and trusted their respective unit to have strict measures in place to prevent cross‐infection. They would have preferred to have surgery without delay despite the potential risk of COVID‐19 (72%). Furthermore, most would have preferred a face‐to‐face follow‐up (59%) while a clear majority did not feel that a routine follow‐up scan should be delayed pending vaccine development (90%).

Our survey shows that, despite the potential risk of COVID‐19, patients are more worried about their health from the underlying aortic pathology than the possibility of contracting COVID‐19. As this was a simple cross‐sectional survey on a small sample, results should be interpreted carefully; larger qualitative studies are needed to understand the impact of the COVID‐19 pandemic and its associated delays over patients who are yet to have aortovascular surgery.

## 6 OUTPATIENTS

To minimize the risk of transmission of COVID‐19 to our patients and health care professionals, all elective face‐to‐face out‐patient reviews were canceled and turned into a virtual consultation by telephone or video‐link. During this COVID‐19 pandemic, telemedicine has been explored and utilized in many other specialities and it has proven its value in such prospect.^20^, ^21^, ^22^ With the passage of the peak of COVID‐19, “face to face” consultations were re‐established for a proportion of patients, with all necessary preventative measures.

## 7 RESTARTING SERVICES

Pathways and standard operating procedures have evolved through multiple iterations in an attempt to return to normal pre‐COVID activity levels while maintaining patient and staff safety. With the advent of effective separation of preoperative and postoperative patients, rapid COVID swabbing, radiological screening, and preoperative patient shielding, we have approached 80% theater operating capacity. Staff safeguarding is maintained through mandatory social distancing, virtual MDTs, face masks as well as track and trace methods. Today, most of the cardiac surgery centers across the UK have resumed their activity in a phased return, initially with urgent/emergency cases. Now, elective cases are considered for surgery on the basis of RCS guidelines and patient self‐isolation for 14 days before admission.

## 8 THE NEW NORMAL AND SUMMARY

Without a doubt, the COVID‐19 pandemic has caused significant disruption to services globally. One of the most affected sectors was health care provision, which required extensive support from governments and volunteers in order for it to provide safe care. Globally there has been service reconfiguration, with some units shutting down some of their services to cope with COVID‐19 patient influx while others diverted their activity into a more centralized, regional hub to be able to deliver emergency, specialist services. NHS England has been in the frontline combating COVID‐19 and cardiothoracic surgical services have been modified to reflect this. In England, the only regions with a clear cardiothoracic surgical pathway for the COVID‐19 pandemic were London through PLEC and the North‐West of England.

Following service modifications, there remain thousands of patients affected by the cancellation of their operation, clinic assessment, or follow‐up. The outcomes of this cohort are unknown; it will be of great interest to understand how these patients, and their quality of life, have been affected.

There is uncertainty about when a full cardiothoracic service will re‐run and whether preoperative testing for COVID‐19 will be a permanent requirement; data are emerging on a daily base and to‐date there are more than 47,000 entries in PubMed. gov related to COVID‐19, increasing on a daily base. Will the present state of affairs be the new norm for cardiac surgeons for the foreseeable future? How will the NHS provide its services in the future? Time and further research will address these questions.

## 9 CONCLUSIONS

The COVID‐19 pandemic has had a significant impact on our nation causing death, disability, and resulting in incalculable effects on families and social structures through long‐lasting consequences for our economy. Through changes in social behavior, the building of our bed base, and changes in NHS structures and priorities, the country stopped the pandemic overwhelming our critical care capacity. There was, however, a significant impact on NHS health care provision including cardiac surgery. The burden of cardiovascular disease on morbidity and mortality, as a consequence of these arrangements, remains unknown at present. Our patient survey showed that patients are more worried about risks to their health from underlying aortic pathology than contracting COVID‐19 in hospital and its associated perioperative risks. Cardiac surgeons have learned an enormous amount on how to manage the service in the context of a national pandemic. Hopefully, this manuscript will offer some insight on how we managed the challenge.

## CONFLICT OF INTERESTS

The authors declare that there are no conflict of interests.

## Supporting information

Supporting information.

Supporting information.

Supporting information.

## Appendix

These are local amendments to freely accessible documents published on the Society of Cardiothoracic Surgery in UK and Ireland website (https://scts.org/covid-19/)

1) North‐West Urgent Cardiothoracic Service
2) Liverpool Heart and Chest Hospital (LHCH) Patient Pathway
3) LHCH Operative standard operating policy

## References

[1] World Health Organization . Naming the coronavirus disease (COVID‐19) and the virus that causes it. 2020. https://www.who.int/emergencies/diseases/novel-coronavirus-2019/technical-guidance/naming-the-coronavirus-disease-(covid-2019)-and-the-virus-that-causes-it. Accessed August 22, 2020.

[2] Dong E , Du H , Gardner L. An interactive web‐based dashboard to track COVID‐19 in real time. Lancet Infect Dis. 2020;20(5):533‐534. 10.1016/S1473-3099(20)30120-1 32087114 PMC7159018

[3] Avery J , Bloom B. COVID‐19, a UK perspective. Eur J Emerg Med. 2020;27(3):156‐157. 10.1097/MEJ.0000000000000700 32224712 PMC7202099

[4] Mohamed Abdel Shafi A , Hewage S , Harky A. The impact of COVID‐19 on the provision of cardiac surgical services. J Card Surg. 2020;35(6):1295‐1297. 10.1111/jocs.14631 32419218 PMC7276906

[5] Royal College of Surgeons of England . Guidance for surgeons working during the COVID‐19 pandemic. https://www.rcseng.ac.uk/coronavirus/joint-guidance-for-surgeons-v1/. Accessed August 22, 2020.

[6] Royal College of Surgeons of England . Clinical guide to surgical prioritisation during the coronavirus pandemic. https://www.rcseng.ac.uk/coronavirus/surgical-prioritisation-guidance/. Accessed August 22, 2020.

[7] Royal College of Surgeons of England . Recovery of surgical services during and after COVID‐19. https://www.rcseng.ac.uk/coronavirus/recovery-of-surgical-services/. Accessed August 22, 2020.

[8] Society for Cardiothoracic Surgery in Great Britain and Ireland (SCTS) . COVID‐19: Recent Publications and Guidance for Cardiothoracic Surgical Practitioners. https://scts.org/covid-19/. Accessed August 22, 2020.

[9] Society for Cardiothoracic Surgery in Great Britain and Ireland (SCTS) . Current Recommendations Regarding Pre‐Operative COVID‐19 Screening CT Scan in Patients Undergoing Cardiothoracic Surgery. https://scts.org/wp-content/uploads/2020/05/SCTS-Current-Recommendations-Regarding-Pre-Operative-COVID-19-Screening-CT-Scan-in-Patients-Undergoing-Cardiothoracic-Surgery-19th-May-2020.pdf. Accessed August 22, 2020.

[10] NHS . Clinical guide for the management of cardiothoracic surgery patients during the Coronavirus pandemic. https://www.england.nhs.uk/coronavirus/wp-content/uploads/sites/52/2020/03/specialty-guide-cardiothoracic-surgery-v1-20-march-2020.pdf. Accessed August 22, 2020.

[11] NHS . Clinical guide for the management of remote consultations and remote working in secondary care during the coronavirus pandemic. https://www.england.nhs.uk/coronavirus/wp-content/uploads/sites/52/2020/03/C0044-Specialty-Guide-Virtual-Working-and-Coronavirus-27-March-20.pdf. Accessed August 22, 2020.

[12] Shafi AMA , Atieh AE , Harky A , Sheikh AM , Awad WI . Impact of COVID‐19 on cardiac surgical training: Our experience in the United Kingdom. J Card Surg. 2020;35(8):1954‐1957. 10.1111/jocs.14693 32557905 PMC7323376

[13] Harky A , Chen R , Pullan M. Examining the impact of COVID‐19 on cardiac surgery services: The lessons learned from this pandemic. J Card Surg. 2020;35:2364‐2366. 10.1111/jocs.14783 32643830 PMC7362009

[14] Hussain A , Balmforth D , Yates M , et al. The Pan London Emergency Cardiac Surgery service: Coordinating a response to the COVID‐19 pandemic. J Card Surg. 2020:1‐7. 10.1111/jocs.14747 PMC7361315 32598501

[15] Zaim S , Chong JH , Sankaranarayanan V , Harky A. COVID‐19 and multiorgan response. Curr Probl Cardiol. 2020;45(8):100618. 10.1016/j.cpcardiol.2020.100618 32439197 PMC7187881

[16] Khan IH , Zahra SA , Zaim S , Harky A. At the heart of COVID‐19. J Card Surg. 2020;35(6):1287‐1294. 10.1111/jocs.14596 32369872

[17] Shafi AMA , Shaikh SA , Shirke MM , Iddawela S , Harky A. Cardiac manifestations in COVID‐19 patients—A systematic review. J Card Surg. 2020;35(8):1988‐2008. 10.1111/jocs.14808 32652713 PMC7404674

[18] Vascular Society . COVID‐19 virus and vascular surgery. https://www.vascularsociety.org.uk/_userfiles/pages/files/Newsletters/2020/Presidents%20update%2027_03_20.pdf. Accessed August 22, 2020.

[19] American College of Surgeons . COVID‐19 guidelines for triage of vascular surgery patients. https://www.facs.org/covid-19/clinical-guidance/elective-case/vascular-surgery. Accessed August 22, 2020.

[20] Hollander JE , Carr BG . Virtually perfect? Telemedicine for COVID‐19. N Engl J Med. 2020;382(18):1679‐1681. 10.1056/NEJMp2003539 32160451

[21] Leung MST , Lin SG , Chow J , Harky A. COVID‐19 and oncology: service transformation during pandemic [published online ahead of print, August 18, 2020]. Cancer. 10.1002/cam4.3384 PMC7461476 32810386

[22] Shirke MM , Shaikh SA , Harky A. Tele‐oncology in the COVID‐19 era: the way forward? Trends Cancer. 2020;6(7):547‐549. 10.1016/j.trecan.2020.05.013 32487487 PMC7250759

