# Supplementary material for: COVID‐19 and cardiac surgery: A perspective from United Kingdom
Source: J Card Surg. 2020 Sep 27;36(5):1649–58. doi: 10.1111/jocs.15039 (PMC7537188; doi:10.1111/jocs.15039)
Supplement: Supplementary file 1 — Supporting information. [file JOCS-36-1649-s001.pdf]

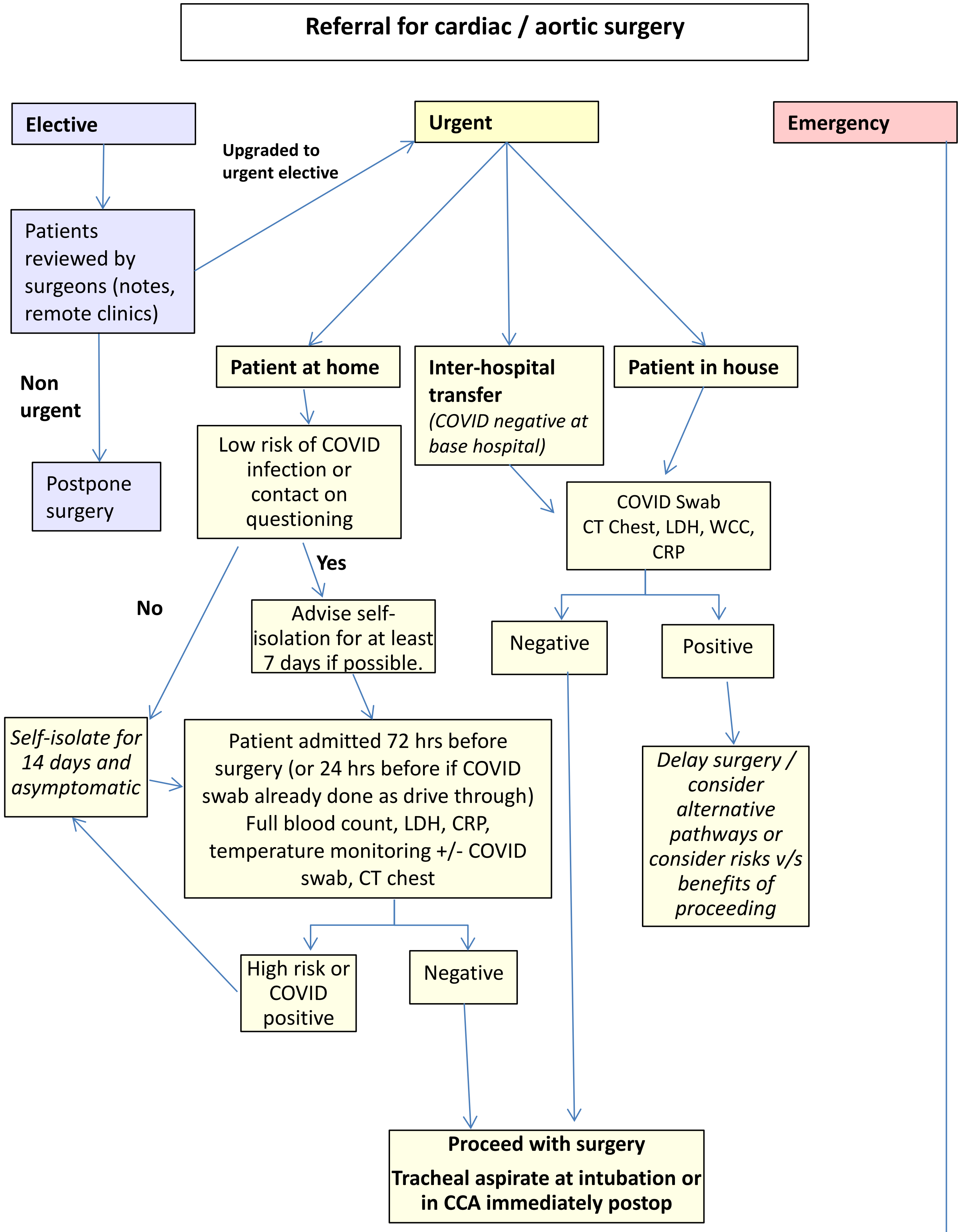

See next page

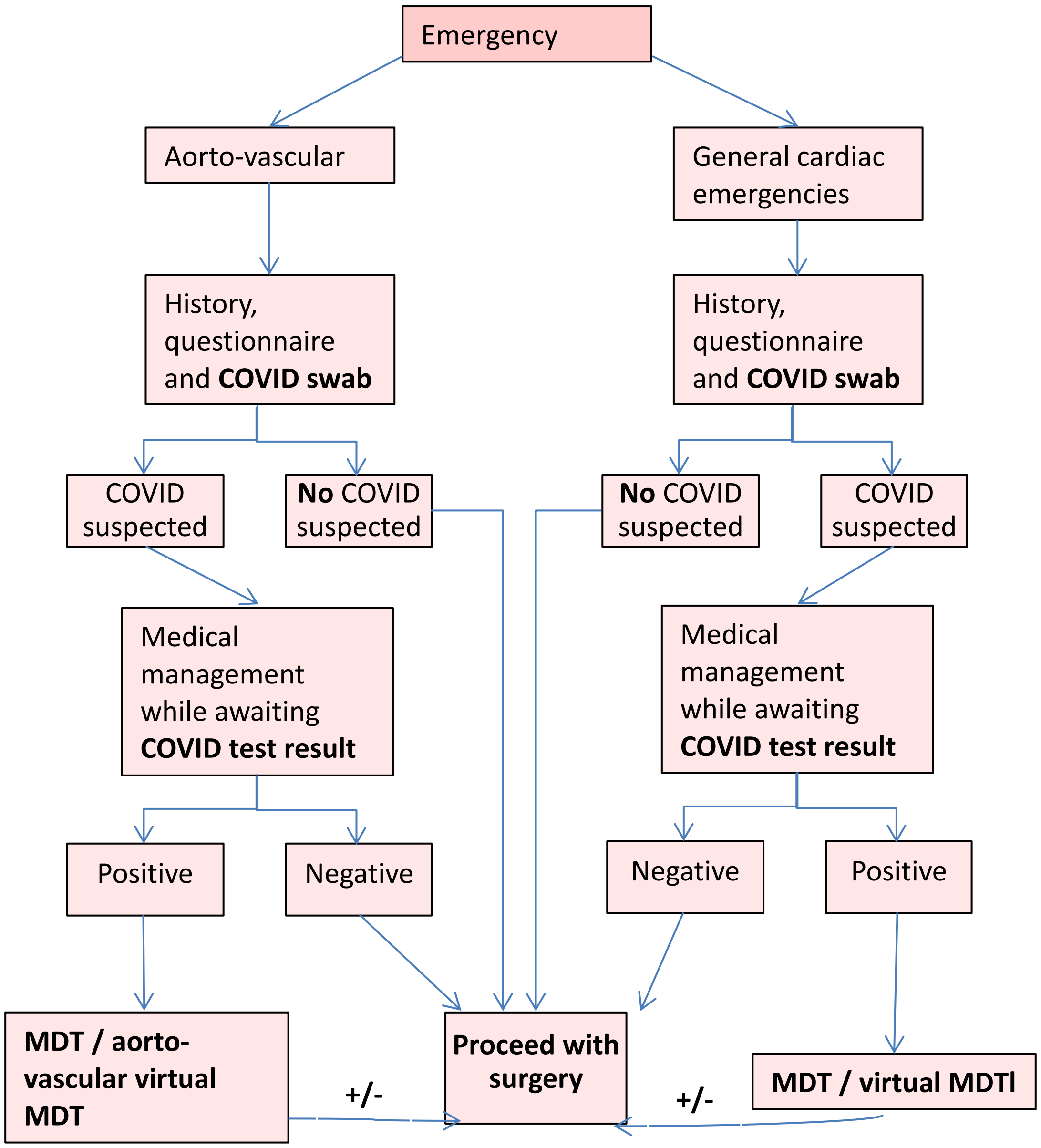

For some emergency patients it might not be possible to wait for screening results to be back before surgery, and it might not be possible to fill in questionnaire if unconscious/ intubated – those will be treated like suspected COVID-positive

Processes for patients whose surgery has been postponed due to a positive COVID swab or CT have

This is for asymptomatic patients who just have a positive test for COVID. Steps as below please. Other permutations and combinations of positive / negative tests should be possible to deal with along the same principles.

Steps after swab / CT comes back as positive

- Ensure baseline D-Dimers, CRP and LDH are done prior to sending home (if they are at home at this point, will need to come in for these tests to OPD)
- Defer surgery for 30 days if possible. If not, minimum of 14 days of self-isolation at home
- Check for symptom status during the intervening period on the phone
- If symptomatic typical of COVID, may need to defer further
- If asymptomatic during the period, do the following tests again (OPD or inpatient)
    - COVID swab for PCR
    - CT Chest for COVID Screening
    - LDH
    - WCC
    - D Dimer
    - CRP

Interpretation of these tests:

- COVID Swab for PCR should be negative
- CT chest findings, if positive before, should be improving or at least stable with no deterioration (CT findings may take a while to resolve)
- LDH, D Dimer and CRP should not show a deterioration compared to baseline
- No evidence of Lymphopenia on WCC

If the above are satisfactory, to proceed with surgery. If not, preferable to defer further if possible or else risk assess each patient individually.

Value of BAL as a separate procedure prior to surgery after first postponement in an asymptomatic patient was discussed. It was felt to represent an added procedure under anaesthetic with little value as the results would be confounding and difficult to interpret if positive (patients could have dead virus with positive PCR test in their samples well past their infected or infective phase and this could take a few weeks to become negative and BAL is very sensitive in picking this up). Patients may be deferred from important surgery on tenuous grounds, not in their best interests.

LHCH COVID Preoperative Pathway for LHCH urgent elective patients

Each cardiac surgeon to choose suitable patients from their own waiting lists on balance of risk

↓

MDT discussion not mandated but can be had where deemed necessary (patients will mostly have been extensively triaged prior to this)

↓

Surgeon to speak to patient on phone to ensure lack of COVID suspicious symptoms and no contact with persons with such symptoms and offer them surgery after explaining reasonable balance of risk

↓

PAs will arrange for TCI of patient as appropriate (24 hours or 72 hrs prior to surgery depending on COVID swab being done as drive through or not

↓

Preop COVID 19 screening

COVID throat swab / CT Chest without Contrast / Serum LDH, CRP

Points to note:

All patients/relatives to be informed that from point of admissions the no visitors rule will apply. This will be the responsibility of the hospital coordinator for urgent transfers and the medical secretary for the elective patients

All tests on admission should be done within 3 days of the date of the operation. Tests requested earlier than this will not be appropriate (as per Pre-Op pathway)

Turnaround time for COVID swabs is 48 hours and improving potentially to 24 hrs soon
