# Supplementary figures and images for: COVID‐19 and cardiac surgery: A perspective from United Kingdom
Source: J Card Surg. 2020 Sep 27;36(5):1649–58. doi: 10.1111/jocs.15039 (PMC7537188; doi:10.1111/jocs.15039)

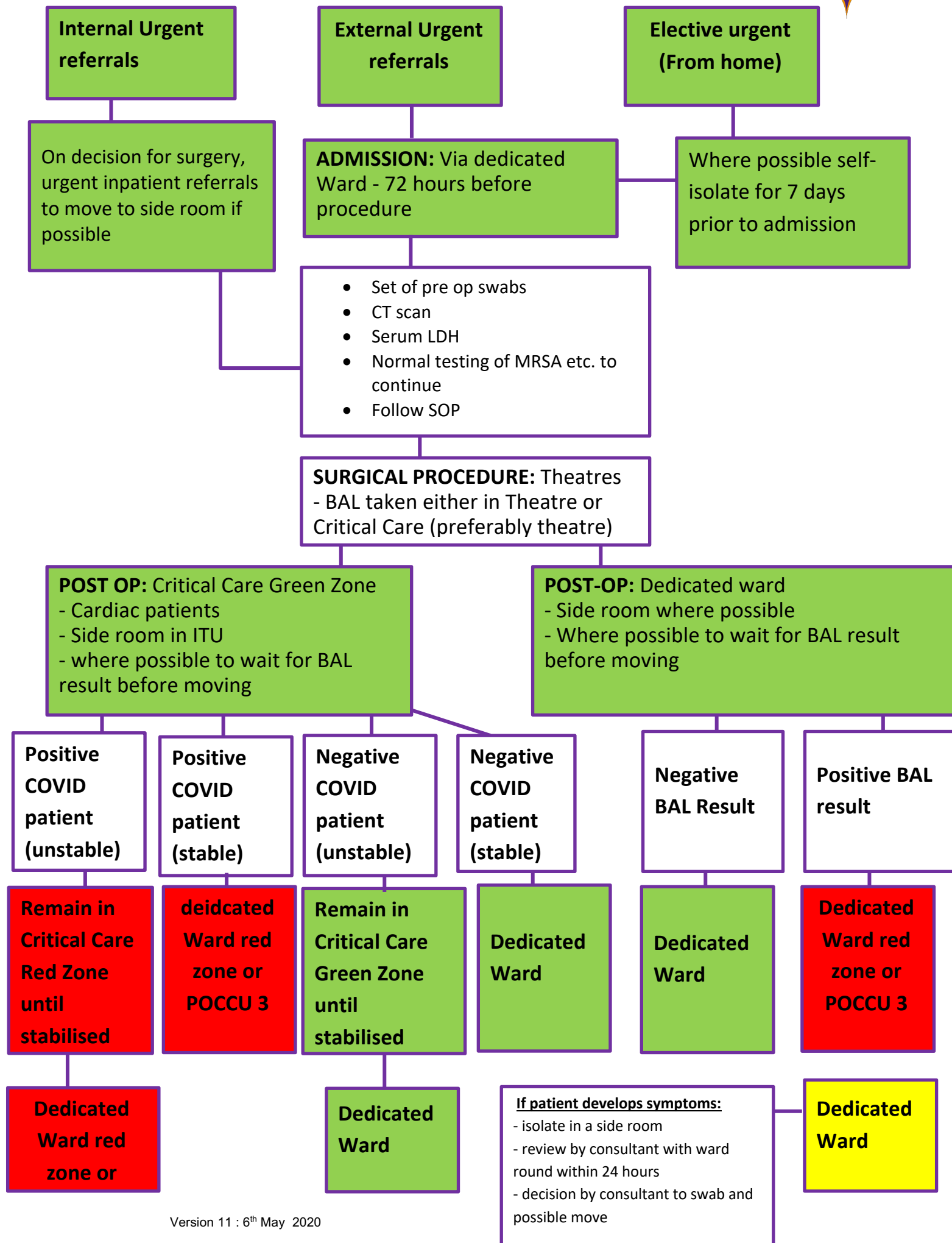

Supplement: Supplementary file 2 — Supporting information. [file JOCS-36-1649-s003.pdf]
