# Supplementary material for: COVID‐19 and cardiac surgery: A perspective from United Kingdom
Source: J Card Surg. 2020 Sep 27;36(5):1649–58. doi: 10.1111/jocs.15039 (PMC7537188; doi:10.1111/jocs.15039)
Supplement: Supplementary file 3 — Supporting information. [file JOCS-36-1649-s002.pdf]

Liverpool Heart and Chest Hospital NHS Foundation Trust

COVID 19 Pandemic

Theatres Standard Operating Procedures (SOP):

This SOP is set to cover theatre practice during the period of COVID 19 pandemic. The following SOP is to be followed for all patients including patients with a negative COVID 19 status to protect all staff working within the theatre complex.

For patients with confirmed and active COVID 19 infection, the following added precautions are required in addition to this SOP:

- Patient to be transferred with a surgical facemask if possible
- Patient to be transferred straight into theatre
- Patient to be anaesthetised and recovered in theatre
- Level of PPE is as for Aerosol Generating Procedures (AGPs) for all staff members within the theatre
- Clear signs on the theatre doors stating “COVID +ve or Suspected Status”
- Due to the theatre air flow cascade (theatre to anaesthetic room), anaesthetic room should remain empty during AGPs in theatre and 20min after the end of AGP

Environment preparation:

Donning location:

Donning for intubation or other anaesthetically related AGPs is to be performed in theatre corridor. Surgical and scrub team can don in the existing scrubbing area.

Donning requirement and location may vary depending on the urgency of the surgical case and patient status, therefore, WHO team brief must include donning timing and location for each member of the team involved.

Doffing location:

Cardiac cases:

In ICU after safe transfer and connection to ICU ventilator

In theatre complex for team members not involved in patient's transfer

Donning and Doffing Technique:

Please refer to Public Health England Guidelines [6]

Theatre ventilation:

It is recommended that ventilation in both laminar flow and conventionally ventilated theatres should remain fully on during surgical procedures where patients may have COVID-19 infection. Air can bypass filtration if a respirator is not fitted perfectly or becomes displaced during use. Those closest to aerosol generation procedures are most at risk. The rapid dilution of these aerosols by operating theatre ventilation will

protect operating room staff. Air passing from operating theatres to adjacent areas will be highly diluted and is not considered to be a risk.

Anaesthetic rooms:

- All anaesthetic rooms are to be stripped of all non critical intubation equipment
- All surfaces to be cleared – Surfaces must be easily wipeable
- Airway trolley to be checked daily and kept just outside the anaesthetic room during the period of intubation. Ideally no USS etc. until after intubation
- One Bougie to be easily available in each anaesthetic room
- As per current practice, difficult airway trolley can be requested but ideally kept outside the anaesthetic room
- HME filters to be inserted at the patient's catheter mount and the expiratory limb of the ventilator circuit
- Any equipment contaminated with respiratory secretions that is not properly cleanable should be disposed of
- Inco-pad to be placed under all patients head to catch respiratory secretions, then safely disposed of ASAP
- Sterillium hand scrub can be used over the donning layer prior to wearing sterile gown and glove for CVP line insertion.

Routine WHO Team Brief:

- To be conducted as per current practice

- Surgery to be rationalised and performed by an experienced surgeon to minimise procedural time as much as possible
- Discuss the level and timing of PPEs for each member of the team.
- Identify any Aerosol Generating Procedures (AGP) expected and when:
    - Intubation, extubation and related procedures, for example, manual ventilation and open suctioning of the respiratory tract (including the upper respiratory tract)
    - Tracheotomy or tracheostomy procedures (insertion or open suctioning or removal)
    - Surgery and postmortem procedures involving high-speed devices. This includes sternotomy during cardiac surgery. For patients with a confirmed COVID 19 -ve status, standard PPE are deemed to be adequate based on the current published evidence supporting that viral detection in blood samples 1%[1]. For patients with COVID 19 +ve or unknown status, full PPE for AGP are mandated (High risk AGP).
    - non-invasive ventilation (NIV); Bi-level Positive Airway Pressure Ventilation (BiPAP) and Continuous Positive Airway Pressure Ventilation (CPAP)
    - High Frequency Oscillatory Ventilation (HFOV)
    - Induction of sputum
    - High flow nasal oxygen (HFNO)

- Insertion or removal of transoesophageal echocardiogram probe
- Cardiac surgery involving (Internal Mammary Artery) IMA harvest or Redo cardiac surgery

Personnel and level of PPE

Personnel to be kept to minimum at all times to minimise risk of viral transmission to staff. If staff change is required for break purposes, it should be planned prior to start of the procedure. See table 1 below

Table 1. Staff level for each procedure

Intubation	Surgery		Extubation	Transfer to ICU
Full PPE for AGP	Routine Surgery with no expected high risk AGPs † standard PPE	*Surgery with expected high risk AGPs Full PPE for AGPs	Full PPE for AGP	† standard PPE
Anaesthetist	Anaesthetist	Anaesthetist	Anaesthetist	Anaesthetist
ODP	Scrub Nurse	Scrub Nurse	ODP	ODP
Runner	Runner	Runner	Runner in anaesthetic room	Surgeon
	Surgeon	Surgeon		Portering staff
	ODP	ODP		
	Perfusionist	Perfusionist		

*** Cardiac Surgery involving IMA, open pleural spaces or Redo surgery with risk of lung injury**

† Routine surgical barrier precautions - Fluid Resistant Surgical Mask, Eye protection, Gowns or Disposable Plastic Apron and Gloves

LHCH COVID - 19 Theatre Intubation Protocol

Your Safety Is the Priority - If unsafe DO NOT Approach

Personnel

Intubator - Most Experienced / Team Leader	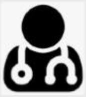
Operating Department Practitioner - ODP	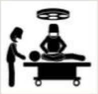
Runner - Outside the intubation room	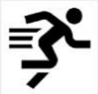

Briefing

Patients details / Allergies / Weight / History	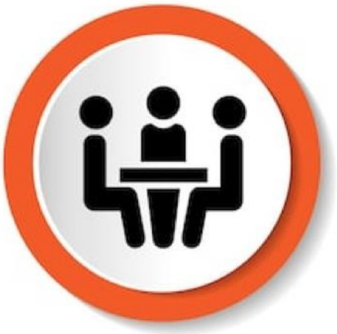
Assign roles and talk through the plan	
Confirm adequate vascular access	
PPEs and Avoidance of contamination of surfaces 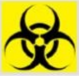	
Agree clinical parameters and interventions	

Drugs

Induction agent - As per Anaesthetist's Choice	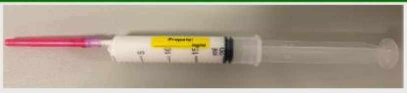
Muscle Relaxant - As per Anaesthetist's Choice	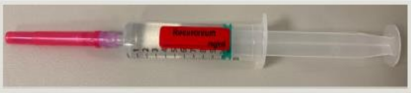
Vasopressor - Phenylephrine 50μg/ml in 20ml	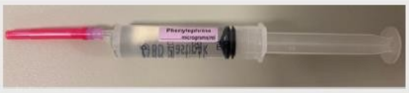
Ephedrine - 3mg/ml in 10ml	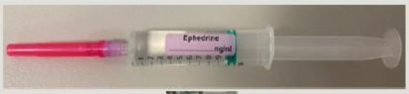
Plastic Tray to be placed on top of Wheeled Sharps bin	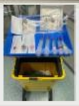
Drugs selection and requirement are at the discretion of the Anaesthetist. The above is an example	

Equipment	
Face mask / HME / Viral Filter / Guidel	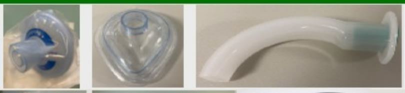
Laryngoscope / Bronchoscope	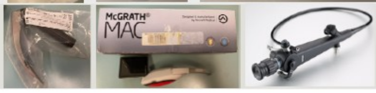
Catheter mount	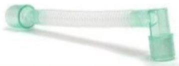
Working Suction / NG tube / Magill Forceps	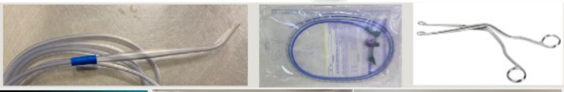
ETT / DLT appropriate size, cut 2cm longer to facilitate clamping	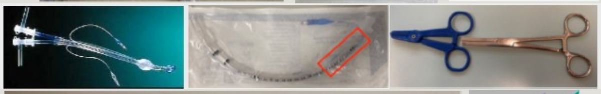
Bougie or Cook airway exchange catheter	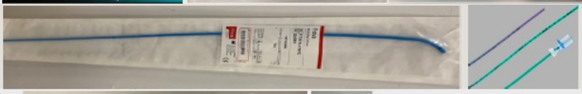
Air syringe / tie / Tape	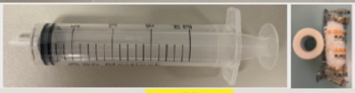
Designate a Tray as a Contamination Zone	

PPE	
Full PPE - FFP3 masks / Visor / Gown	
Double Gloves + Extra	

INTUBATION PROTOCOL - Theatre

Prepare ventilator settings before touching the patient

Pre-oxygenate +2min AND optimise Haemodynamics

Intubator maintains airway | **ODP Prepares for intubation**

Maintain Situational Awareness - Vital Signs | **Obtains extra equipment if required**

AVOID hand ventilation - if required use 2 hands 2 persons

Used Laryngoscope / Yanker Suction / Outer Gloves - PLACED IN Contamination Tray / Zone

After successful intubation AND connection to HME / Viral Filter contamination risk is significantly reduced

Insert TOE Probe while in the anaesthetic room - TOE probe insertion is considered as AGP

Surgery:

- Maintain staffing level to minimum
- Minimise opening doors during surgery and use telephone if communication with the team is required during the procedure
- If ventilation is required to be suspended (Sternotomy or Surgical Request), use ManSpont mode and **KEEP ALARMS ON** at all times. Announce, **"Ventilator OFF"**. At the end of surgical requirement, restart ventilation and announce, **"Ventilator ON"**, ensure normal CO2 trace on ventilator display.
- Repeat COVID 19 BAL sample to be taken for all surgical patients after intubation:
    - Cardiac patients:
        - Anaesthetic team to attach a closed suction catheter mount system at time of intubation (Stericath). Instil 20ml of Saline, wait for 1min and aspirate in a sputum sampling pot
        - Sample to be handed to scrub team to be labelled and sent to the lab
- AGP precautions are advisable during IMA harvest or redo surgery to minimise the risk of viral transmission from potential breach of the lung

End of surgery:

- Complete WHO sign off check as per current practice
- Prepare for transfer to ITU as per current practice
- No change to current monitoring and ventilatory support standards. HME filter and Mapleson C - Circuit can be used
- For added protection when transferring patients with COVID +ve or unknown status, a clear plastic sheet can be used to cover the patient's head and breathing circuit (can be obtained from critical care)
- Minimise personnel for transfer
  1. Anaesthetist
  2. ODP
  3. Surgeon
  4. Portering staff

Cleaning and decontamination (Public Health England) [7]:

Clearance of contaminated aerosols is dependent on the ventilation and air change within the room.

Following discharge from the theatre room the following procedure must be observed if the patient was extubated in theatre for all cases:

- Doors fully closed and the room left vacant for a minimum period of 20mins

- Cleaning and decontamination performed by trained staff wearing standard PPE (As per table 1)

- It is recommended following cleaning with a neutral detergent, a chlorine based disinfectant is used, in accordance with the local infection prevention control team

- Dedicated or disposable equipment (such as mop heads, cloths) must be used for environmental decontamination. Reusable equipment (such as mop handles, buckets) must be decontaminated after use with a chlorine-based disinfectant as described above. Communal cleaning trollies should not enter the room.

- For patients who are transferred out of theatre and remain intubated / ventilated via an HME protected filter (e.g Cardiac patients), the above 20min pause is not required unless there was an AGP (e.g Bronchoscopy) with 20min of leaving the theatre. This needs to be clarified by the consultant anaesthetist looking after the patient

Clearance of aerosols is dependent on the ventilation and air change within the room. Once an end to dispersion can be defined (such as the patient leaving the room), a single air change is estimated to remove 63% of airborne contaminants and similarly with each subsequent air change. After 5 air changes, less than 1% of the original airborne contamination is thought to remain.

In an isolation room with 10 to 12 air changes per hour (ACH) a minimum of 30 minutes will reduce contamination to less than 1%. In a side room with 6 ACH, one hour would be a pragmatic time, allowing for aerosols settling out as well as being removed by ventilation.

Following transfer (recovery) and/or discharge of the patient, it is recommended that the room is left vacant with the door closed for 20 minutes in a negative pressure isolation room or one hour for a neutral pressure room prior to performing a terminal clean. Windows to the outside in neutral pressure rooms can be opened.
